# Supplementary material for: Attentional shifts bias microsaccade direction but do not cause new microsaccades
Source: Commun Psychol. 2024 Oct 22;2:97. doi: 10.1038/s44271-024-00149-7 (PMC11496105; doi:10.1038/s44271-024-00149-7)
Supplement: Supplementary file 2 — Reporting Summary [file 44271_2024_149_MOESM2_ESM.pdf]

nature portfolio

Corresponding author(s): Baiwei Liu & Freek van Ede
Last updated by author(s): Jul 10, 2024

Reporting Summary

Nature Portfolio wishes to improve the reproducibility of the work that we publish. This form provides structure for consistency and transparency in reporting. For further information on Nature Portfolio policies, see our Editorial Policies and the Editorial Policy Checklist.

Statistics

For all statistical analyses, confirm that the following items are present in the figure legend, table legend, main text, or Methods section.

n/a	Confirmed	
☐	☒	The exact sample size (*n*) for each experimental group/condition, given as a discrete number and unit of measurement
☐	☒	A statement on whether measurements were taken from distinct samples or whether the same sample was measured repeatedly
☐	☒	The statistical test(s) used AND whether they are one- or two-sided<br>*Only common tests should be described solely by name; describe more complex techniques in the Methods section.*
☐	☒	A description of all covariates tested
☐	☒	A description of any assumptions or corrections, such as tests of normality and adjustment for multiple comparisons
☐	☒	A full description of the statistical parameters including central tendency (e.g. means) or other basic estimates (e.g. regression coefficient) AND variation (e.g. standard deviation) or associated estimates of uncertainty (e.g. confidence intervals)
☐	☒	For null hypothesis testing, the test statistic (e.g. *F*, *t*, *r*) with confidence intervals, effect sizes, degrees of freedom and *P* value noted<br>*Give P values as exact values whenever suitable.*
☐	☒	For Bayesian analysis, information on the choice of priors and Markov chain Monte Carlo settings
☒	☐	For hierarchical and complex designs, identification of the appropriate level for tests and full reporting of outcomes
☐	☒	Estimates of effect sizes (e.g. Cohen's *d*, Pearson's *r*), indicating how they were calculated

Our web collection on statistics for biologists contains articles on many of the points above.

Software and code

Policy information about availability of computer code

Data collection	python 3.8 with psychopy 2022;EyeLink 1000
Data analysis	FieldTrip version 20201023; Matlab version 2022a; Relevant code associated with microsaccade detection is shared through OSF.

For manuscripts utilizing custom algorithms or software that are central to the research but not yet described in published literature, software must be made available to editors and reviewers. We strongly encourage code deposition in a community repository (e.g. GitHub). See the Nature Portfolio guidelines for submitting code & software for further information.

Data

Policy information about availability of data

All manuscripts must include a data availability statement. This statement should provide the following information, where applicable:
- Accession codes, unique identifiers, or web links for publicly available datasets
- A description of any restrictions on data availability
- For clinical datasets or third party data, please ensure that the statement adheres to our policy

All data will be made publicly available before publication.

Human research participants

Policy information about studies involving human research participants and Sex and Gender in Research.

Reporting on sex and gender	Twenty-five healthy human volunteers from Vrije Universiteit Amsterdam participated in the study (age range: 18 - 44; 5 male and 20 female; 25 right-handed; 5 corrected-to-normal vision: 1 glasses and 4 lenses).
Population characteristics	see above
Recruitment	see above
Ethics oversight	Experimental procedures were reviewed and approved by the local Ethics Committee at the Vrije Universiteit Amsterdam.

Note that full information on the approval of the study protocol must also be provided in the manuscript.

Field-specific reporting

Please select the one below that is the best fit for your research. If you are not sure, read the appropriate sections before making your selection.

☐ Life sciences ☒ Behavioural & social sciences ☐ Ecological, evolutionary & environmental sciences

For a reference copy of the document with all sections, see nature.com/documents/nr-reporting-summary-flat.pdf

Behavioural & social sciences study design

All studies must disclose on these points even when the disclosure is negative.

Study description	We used a computer and an eyetracker to collect quantitative data
Research sample	Twenty five students from Vrije Universiteit Amsterdam, with age range: 18 - 44; 5 male and 20 female; 25 right-handed; 5 corrected-to-normal vision: 1 glasses and 4 lenses
Sampling strategy	Sample size of 25 was determined a-priori based on previous publications from the lab with similar experimental designs that relied on the same outcome measure
Data collection	We used a computer and an eyetracker to collect quantitative data
Timing	December 2022 to March 2023
Data exclusions	One participant was excluded for all analyses due to chance-level performance
Non-participation	No participants dropped out during the experiment
Randomization	We used a within-participant design. Within each participant, both conditions (attention-directing cues and neutral cues) were randomly intermixed within each block as were attention-directing cues to left and right memory items.

Reporting for specific materials, systems and methods

We require information from authors about some types of materials, experimental systems and methods used in many studies. Here, indicate whether each material, system or method listed is relevant to your study. If you are not sure if a list item applies to your research, read the appropriate section before selecting a response.

Materials & experimental systems

n/a	Involved in the study	
☒	☐	Antibodies
☒	☐	Eukaryotic cell lines
☒	☐	Palaeontology and archaeology
☒	☐	Animals and other organisms
☒	☐	Clinical data
☒	☐	Dual use research of concern

Methods

n/a	Involved in the study	
☒	☐	ChIP-seq
☒	☐	Flow cytometry
☒	☐	MRI-based neuroimaging
